# Microcystic Variant of an Intraosseous Meningioma in the Frontal Area: A Case Report

**DOI:** 10.1155/2014/527267

**Published:** 2014-06-17

**Authors:** Jan Bujok, Marek Bienioszek

**Affiliations:** ^1^Department of Neurosurgery, Provincial Hospital, Armii Krajowej 101 Street, 43-316 Bielsko-Biała, Poland; ^2^Faculty of Health Sciences, University of Bielsko-Biała, Willowa 2 Street, 43-300 Bielsko-Biała, Poland; ^3^Department of Pathomorphology, Beskidzkie Oncology Centre, John Paul II Municipal Hospital, Wyzwolenia 18 Street, 43-300 Bielsko-Biała, Poland

## Abstract

Meningiomas located inside the bone of the calvaria belong to the group of rare types of tumours. The microcystic variant is the least common in this area. Due to their similarity to other tumours in this area, the imaging test of those tumours may constitute the source of an improper preoperative diagnosis. According to the records of the Department of Neurosurgery in Bielsko-Biała, 133 patients diagnosed with an intracranial meningioma confirmed by a histopathological test were operated in the last 10 years (2004–2014). In the histopathological test, three patients were diagnosed with the microcystic variant, which constitutes 2.25% of the cases. Only one variant of microcystic meningioma was located inside the bone, which constitutes 0.75% of all the meningiomas operated.

## 1. Introduction

Meningiomas primarily localized in the extrameningeal area constitute approximately 1.6% of tumours with 25% of the tumours being located in the area of the convexity [1]. According to our own records, out of 14 cases of the bone tumours of the calvaria operated in the period from January 2004 to February 2014 at our Department of Neurosurgery, meningiomas constituted 21% of the cases. The microcystic variant of meningioma located inside the bone of the calvaria is very rare [2, 3]. The unique structure of the tissue of this benign tumour, containing cells with cystic contents, causes interpretative difficulties already at the stage of an imaging diagnosis where the tumour needs to be differentiated from other intraosseous pathology.

The following study presents the case of a microcystic meningioma found in a 59-year-old woman who underwent a successful surgical treatment.

## 2. Case Report

A 59-year-old woman was admitted to the Department of Neurosurgery due to a lesion localized inside the bone in the right frontal area, discovered accidentally in the CT (computed tomography) scan of her head performed for another reason. Before the diagnosis, the patient complained about occasional headaches and recent memory disorders.

The medical history did not prove any past head injury or any other pathology. The physical examination showed a proper neurological state.

**Figure 1:**
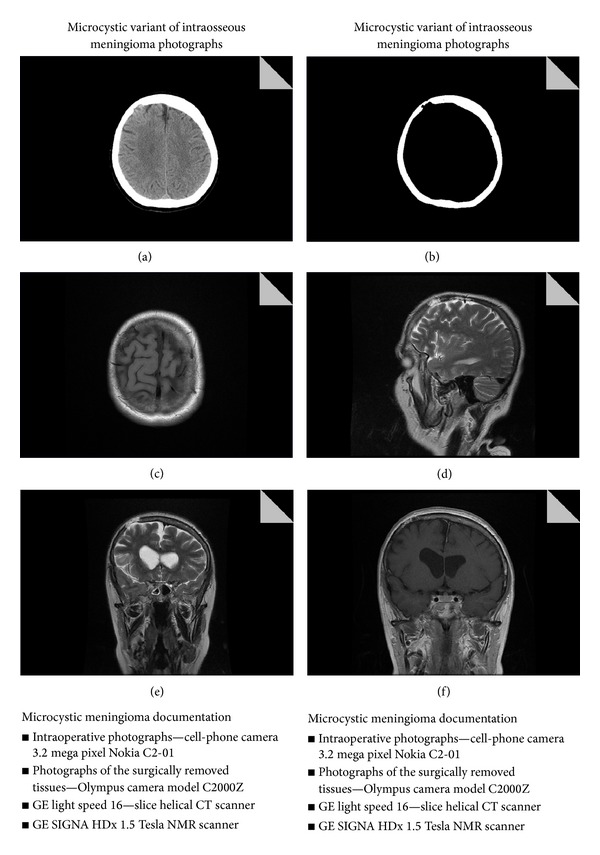
Imaging characteristics: (a) head CT scan, axial projection, and osteolytic defect in the right frontal area in the location of the bone tumour; (b) head CT scan, axial projection, bone window, and osteolytic defect in the right frontal area; (c) MR of the brain, axial projection, T1 SE, and the area of mixed signal intensity inside the bone in the right frontal area; (d) MR of the brain, image in the sagittal plane, and T2 FSE hyperintense area in the right frontal area; (e) MR of the brain, image in the coronal plane, and T2 FSE, hyperintense area in the right frontal area; (f) MR of the brain, image in the coronal projection, T1 following the application of contrast agents, and contrast enhancement features involving only the bone structure.

The performed CT scan of the head revealed blotchy areas of osteolysis in the right frontal area, measuring approximately 24 mm in diameter. The CT examination suggested an eosinophilic granuloma (Figures 1(a)-1(b)). The intracranial mass was not found in the area of the osteolytic defects. The MR (magnetic resonance) imaging showed irregular areas of an increased signal intensity on the T2 weighted image in the area of the calvaria and a mixed signal intensity on the T1 weighted image, with contrast-enhanced features, involving only the structure of the bone (Figures 1(c)–1(f)).

**Figure 2:**
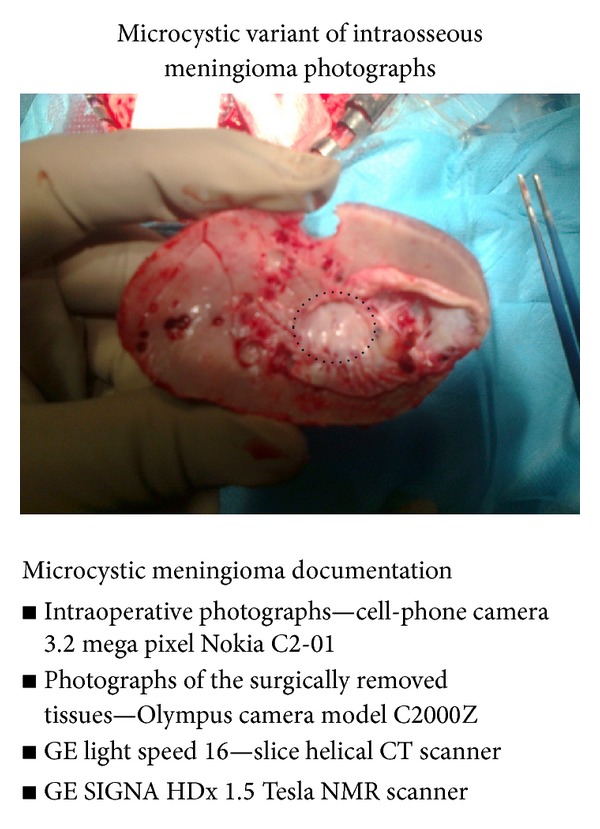
Operative specimen. The internal surface of the bone flap removed with the tumour. The area of the tumour located inside the osteolytic defect of the bone with the adhering part of the dura mater marked with dots.

The ambiguous image suggested the foci of a myeloma, untypical metastases. The results of the laboratory tests were correct. The patient underwent an operation. During the operation bluish discoloration was found on the surface of the bone. After removing the bone flap, a lesion localized inside the bone and adjacent to the dura mater was discovered (Figure 2). There were singular lesions revealed under the dura mater, forming part of the tumour and measuring approximately 2-3 mm in dimension on the surface of the arachnoidea above the area of the bone foramen, which were removed and coagulated, preserving the venous system intact. The bone flap with the tumour and the dura mater were excised. The reconstructive operation of the dura mater and the bone defect was performed with the use of the Neuro-Patch material and the cranioplastic type of material, respectively. After the wound had healed, the patient in the same neurological state as before the operation and without any deficiency signs was discharged from the hospital.

**Figure 3:**
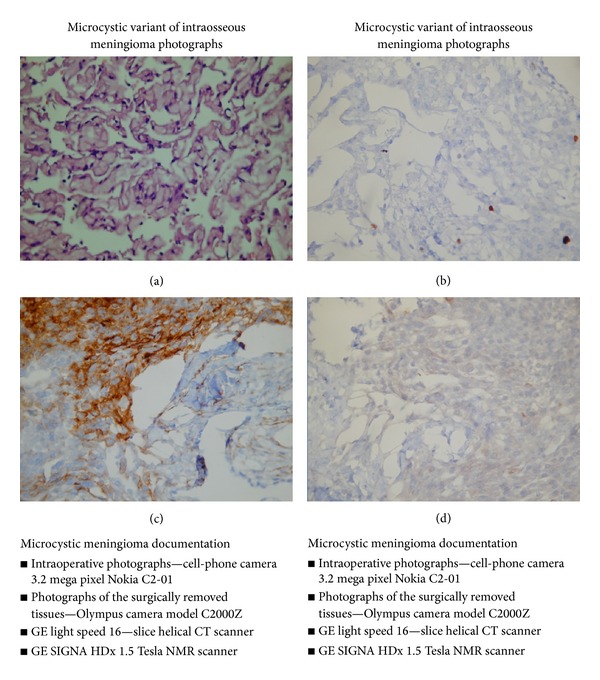
Histopathology: (a) H-E, photomicrograph, original magnification 400x, fragment of the surgically removed tissues, and numerous microcysts containing a pale eosinophilic fluid, surrounded by processes of neoplastic cells; (b) Ki-67, when it is positive in singular cells, it points to very low proliferative activity of the neoplasm, photomicrograph, original magnification 400x, and fragment of the surgically removed tissues; (c) EMA (+), photomicrograph, original magnification 400x, and fragment of the surgically removed tissues; (d) CK (+), photomicrograph, original magnification 400x, and fragment of the surgically removed tissues.

The histopathological test was performed in its final form following additional immunohistochemical tests (EMA (+) (epithelial membrane antigen), CK AE1/AE3 (+) (cytokeratin), Ki 67 (positive in singular cells), CD 31 (+) (cluster of differentiation 31), and CD 34 (+) (cluster of differentiation 34) in the area of blood vessels). The microcystic variant of meningioma was diagnosed (Figures 3(a)–3(d)).

## 3. Discussion

Microcystic variants of meningiomas belong to the group of rare tumours. Most of the studies describing this variant of meningioma are casuistic reports [2, 4–8]. Following their analysis of the professional literature, Ichimura et al. [2] claim the 1.6% frequency of occurrence of microcystic variants of intracranial meningiomas.

Meningiomas are most frequently localized in the intrameningeal area. Tumours which are localized in the extrameningeal area, including the intraosseous area, have been known as ectopic tumours [1, 9]. On the basis of their own case studies and the analysis of the professional literature, Lang et al. [1] named those tumours as primary extradural meningiomas and suggested the classification into type I (purely extracalvarial), type II (purely calvarial), and type III (calvarial with extracalvarial extension). Depending on the localization, type II and type III have been further categorized as C (convexity) and B (skull base).

The studies which described the microcystic variant of meningioma mostly indicated its intrameningeal localization [4, 6–8]. The first literature-based description of the microcystic meningioma located inside the bone of the calvaria was presented by Okamoto et al. in their study [3] in 2000.

According to our own records of the Department of Neurosurgery a total of 133 patients diagnosed with an intracranial meningioma, confirmed by a histopathological test, were operated in the period from January 2004 to February 2014. The microcystic variant was diagnosed in 3 cases, which constitutes approximately 2.25% of the cases and presents similar results to the data collated in the literature [2, 4].

According to our records, one meningioma of the microcystic type was localized inside the bone, which constitutes 0.75% of all the intracranial meningiomas operated.

Our own records also include 14 bone tumours of the cranial vault operated in 2004–2014. The most numerous group of tumours was formed by osteomas (approximately 28%), followed by meningiomas (21%) and other tumours: fibrous dysplasia, metastasis of an adenocarcinoma, multiple myeloma, epidermal cyst, angioma, Langerhans cell histiocytosis, and osteopetrosis (approximately 7% each). Intraosseous meningiomas (3 cases) occurred as the meningothelial variants of meningioma (2 cases) and as the microcystic variant (1 case) described in this study.

The patients were 1 man and 2 women. The average age of the patients was 46 (the range from 25 to 59 years).

The performed CT scan of the head in the case described herein revealed the area of osteolysis localized inside the frontal bone. In the case of the microcystic variant of meningioma located in the diploë, other studies also described some osteolytic lesions [2, 3]. In the case of the intraosseous meningioma, the osteolytic lesions need to be differentiated from such pathologies such as plasmacytoma, giant cell tumor, metastatic cancer, hemangioma, epidermoid cyst, eosinophilic granuloma, fibrous dysplasia, osteogenic sarcoma, aneurysmal bone cyst, and chondroma [10–12].

The studies discussing other histological variants of intraosseous meningiomas observed in the CT examination described both the osteolytic and the osteosclerotic lesions [9–15].

The MR imaging is particularly useful when determining the exact location of the tumour as well as its localization with respect to the dura mater. In the case presented, the MR imaging revealed irregular areas of an increased signal intensity on the T2 weighted image in the area of the calvaria and a mixed signal intensity on the T1 weighted image, with contrast-enhanced features, involving only the structure of the bone. A similar image in the MR imaging of the microcystic variant of an intraosseous meningioma has been provided by other authors [2, 3].

In the case of other variants of intraosseous meningiomas, the MR images are not characteristic and according to other authors they are important in determining the exact location of the lesion and specifying the localization of the tumour with respect to the dura mater [9–12, 14].

In their study involving the retrospective analysis of 16 patients diagnosed with primary extrameningeal meningiomas, who were undergoing treatment in two centres in the period from 1992 to 2004, Bassiouni et al. [9] indicate that the MR imaging revealed contrast enhancement adjacent to the intraosseous meningioma of the dura mater in 11 patients. In turn, the intraoperative examination revealed the tumour infiltration into the dura mater in 14 patients [9].

On the basis of the CT and MR imaging Lang et al. noticed the infiltration of the dura mater in 60% of the cases, confirmed by the intraoperative examination in 40% of the cases [1].

The tissue structure of the microcystic meningioma is characterized by the presence of neoplastic cells. The histopathological diagnosis requires the additional staining of specimens [2–6, 8]. In order to confirm the diagnosis in the case discussed, the additional staining was performed (EMA, CK, Ki 67, CD 31, CD 34) on the basis of which the microcystic variant of meningioma was histopathologically diagnosed.

An intraoperative diagnosis made it possible to reveal a tumour localized inside the bone, with the tumour itself adhering to the external surface of the dura mater, which prevented its effective separation from the tumour. The continuity of the dura mater was in all probability slightly damaged by the tumour, which could be noticed, considering the partial adherence to the arachnoidea and the fact that 2-3 mm elements of the tumour were revealed under the dura mater disseminated on the surface of the arachnoidea virtually above the whole area of the performed craniectomy. In their study related to the meningothelial variant of intraosseous meningioma Kudo et al. [16] noticed the damaged continuity of the dura mater, which they documented in a histopathological test, as well as fine elements of the tumour tissue on the arachnoidea surface.

Similar conclusions concerning the intraosseous meningioma infiltration into the dura mater were reached by Bassiouni et al. [9] who emphasized the significance of the MR imaging in the preoperative diagnosis and suggested their own modification to the classification of primary extradural meningiomas developed by Lang et al. [1].

Our own intraoperative and microscopic analyses suggest that the osteolytic defect of the bone in the place of the tumour and the direct adherence to the external layer of the dura matter in the case presented may be a secondary phenomenon. The meningioma itself is primarily localized in the diploë and a long-term compression or even the cells themselves, which may burst and release their contents outside, in all likelihood provide the mechanism of the expansion of this unique form of meningioma which also spreads beyond the dura mater barrier and is the source of dissemination on the surface of the arachnoidea.

Although the infiltration of the dura mater by the tumour localized in the diploë was not revealed in the preoperative tests, a detailed intraoperative examination is substantiated during the operation and so is the contemplation of removing the dura mater and performing the reconstructive operation in case one suspects an intraosseous meningioma which needs to be taken into account in the differential diagnosis of bone tumours of the cranial vault.
